# More than a sidekick: GIP signalling and cardiovascular outcomes

**DOI:** 10.1093/eurheartj/ehaf944

**Published:** 2025-12-22

**Authors:** Berkan Kurt, Florian Kahles

**Affiliations:** Department of Internal Medicine I—Cardiology, University Hospital Aachen, RWTH Aachen University, Pauwelsstraße 30, D-52074 Aachen, Germany; Department of Internal Medicine I—Cardiology, University Hospital Aachen, RWTH Aachen University, Pauwelsstraße 30, D-52074 Aachen, Germany

## Abstract

**Graphical Abstract:**
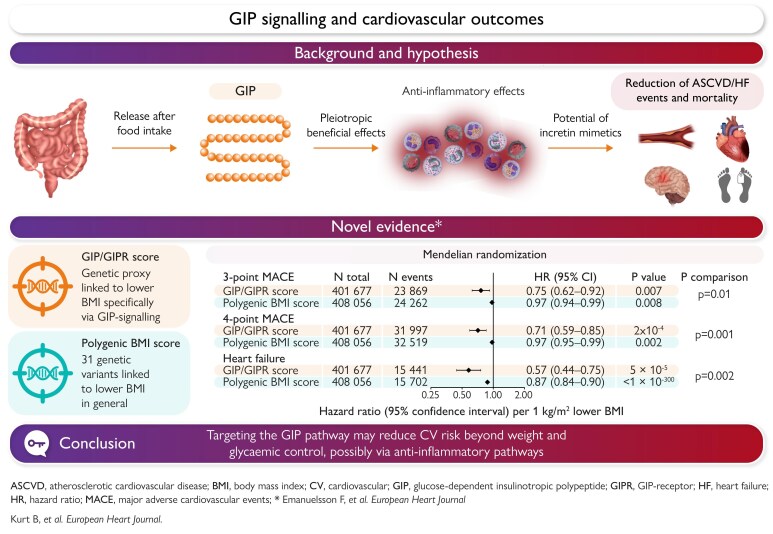
Glucose-dependent insulinotropic polypeptide (GIP), released from the gut after food intake and has pleiotropic beneficial and anti-inflammatory effects, potentially contributing to the effect of incretin mimetics to reduce atherosclerotic cardiovascular disease (ASCVD) and heart failure (HF) events and mortality. Novel evidence from a Mendelian randomization study by Emanuelsson et al. using a GIP/GIP receptor (GIPR) genetic score as a pathway-specific proxy for lower body mass index (BMI), compared with a polygenic BMI score, shows that per 1 kg/m² lower BMI, GIP/GIPR-mediated BMI reduction is associated with greater relative reductions in major adverse cardiovascular events (MACE) and HF than BMI lowering in general. These genetic data suggest that targeting the GIP pathway may reduce cardiovascular risk beyond effects on body weight and glycaemic control, possibly via anti-inflammatory mechanisms

Glucose-dependent insulinotropic polypeptide (GIP), released from the gut after food intake and has pleiotropic beneficial and anti-inflammatory effects, potentially contributing to the effect of incretin mimetics to reduce atherosclerotic cardiovascular disease (ASCVD) and heart failure (HF) events and mortality. Novel evidence from a Mendelian randomization study by Emanuelsson et al. using a GIP/GIP receptor (GIPR) genetic score as a pathway-specific proxy for lower body mass index (BMI), compared with a polygenic BMI score, shows that per 1 kg/m² lower BMI, GIP/GIPR-mediated BMI reduction is associated with greater relative reductions in major adverse cardiovascular events (MACE) and HF than BMI lowering in general. These genetic data suggest that targeting the GIP pathway may reduce cardiovascular risk beyond effects on body weight and glycaemic control, possibly via anti-inflammatory mechanisms

**This editorial refers to ‘Genetic variants of glucose-dependent insulinotropic polypeptide (GIP) signalling as proxy for body weight reduction and cardiovascular risk’, by F. Emanuelsson *et al*., https://doi.org/10.1093/eurheartj/ehaf779.**

Obesity is not a failure of an individual but a life-threatening chronic disease. Approximately more than 1 billion people worldwide live with obesity.^1^ Mortality rises with body mass index (BMI). With a BMI of 30–35 kg/m^2^, median survival is shorter by ∼2–4 years, and at 40–45 kg/m^2^ by ∼8–10 years, which is a reduction of life expectancy comparable with smoking.^2^

Obesity drives metabolic and cardiorenal diseases. The cardiovascular–kidney–metabolic (CKM) syndrome describes a spectrum from preserved cardiometabolic health to established cardiovascular (CV) disease.^3^ Within the CKM syndrome, excess and dysfunctional adipose tissue is the earliest manifestation, which accelerates development of further metabolic risk factors, such as insulin resistance, hypertension, and early chronic kidney disease. Later on, subclinical CV disease appears and is followed by manifestation of coronary and peripheral artery disease, stroke, and heart failure. On this basis, obesity is not only a comorbidity but also an upstream driver that sustains risk across the CKM syndrome. However, because most therapeutic strategies target downstream risk factors while the upstream driver—excess and dysfunctional adipose tissue—often remains untreated, residual risk persists despite implementation of guideline-based prevention strategies in the CKM spectrum.

Management of obesity begins with structured lifestyle interventions aimed at achieving substantial and sustained weight loss, which is required for meaningful improvements of CV outcomes. In Look AHEAD, intensive lifestyle interventions to achieve weight reduction did not reduce CV events in patients with type 2 diabetes mellitus (T2DM) and obesity, suggesting that additional strategies such as pharmacotherapy are needed to reduce CV risk.^4^

While earlier antiobesity agents were limited by tolerability and safety, with incretin-based therapies an effective pharmacological strategy became available in clinical practice. Glucagon-like peptide-1 (GLP-1) is an incretin hormone released from the gut after food intake. GLP-1 receptor agonists (GLP-1RAs) improve glycaemic control and provide clinically meaningful weight loss in patients with T2DM and/or obesity.^5^ Experimental and clinical data also show beneficial pleiotropic effects on inflammation, endothelial function, and plaque stability.^5^ Randomized CV outcome trials (CVOTs) in T2DM consistently report reduction of major adverse CV events (MACE), with complementary evidence in heart failure.^6,7^ Moreover, SELECT demonstrated that beneficial effects on CV outcomes extended beyond T2DM, as treatment with the GLP-1RA semaglutide reduced CV events in non-diabetic individuals with established atherosclerotic CV disease (ASCVD) and obesity.^8^ Furthermore, data from SELECT even suggest that the reduction of CV risk in these individuals might be independent of weight loss.^8^

Co-agonists that target more than one incretin have emerged from this rationale. Tirzepatide, also known as a “twincretin”, is a dual receptor agonist of GLP-1 and GIP (glucose-dependent insulinotropic polypeptide) that lowers glycated haemoglobin (HbA_1c_) and shows greater weight loss compared with GLP-1RA alone.^9^

In heart failure with preserved ejection fraction (HFpEF), often linked to an obese phenotype, randomized trials showed that semaglutide improved symptoms, physical limitations, and exercise capacity.^7^ Moreover, tirzepatide reduced heart failure events, extending the signal from symptom control to hard CV outcomes.^10^ Emulated trials in real-world evidence data even indicate that such drugs could reduce mortality in HFpEF patients.^11^

In this issue of the *European Heart Journal*, Emanuelsson *et al*. use human genetics to investigate whether the GIP pathway contributes to CV risk modification beyond effects on weight and glycaemia.^12^ The authors should be congratulated for this important contribution, which opens up new perspectives on how incretin biology might be harnessed for CV risk reduction. The analysis is based on the UK Biobank for one-sample Mendelian randomization, and on FinnGen for two-sample Mendelian randomization. The authors constructed a weighted GIP/GIP receptor (GIPR) score linked to BMI and/or glycaemic traits and T2DM, and used it as the pathway-specific exposure. As a comparator, they applied a score representing genetic variants associated with BMI with exclusion of GIP/GIPR-related genes (polygenic BMI score). The primary outcomes assessed were 3-point major adverse CV events (3-point MACE), 4-point MACE, and heart failure (*Graphical Abstract*).

Importantly, genetically proxied BMI lowering via the GIP/GIPR score was associated with lower risks of MACE and heart failure. When compared with a 1 kg/m^2^ reduction in BMI, relative risk reduction calculated with the GIP/GIPR score was larger than with the polygenic BMI score. Replication in the FinnGen cohort demonstrated similar results, and sensitivity analyses were consistent. Interestingly, mediation analyses suggested that BMI and HbA_1c_ account for only part of the observed risk reduction. These findings are highlighted by the authors as evidence showing that additional mechanisms may be relevant in CV risk reduction achieved by the GIP pathway.

Systemic low-grade inflammation may provide a plausible explanation for this open question. The GIP/GIPR score was associated with lower high-sensitivity C-reactive protein (hsCRP) and, when hsCRP was accounted for in mediation analyses, CV effects were modestly reduced, suggesting that anti-inflammatory effects add to benefits of GIP beyond weight reduction and glycaemic control. Experimental findings support this interpretation, as GIP reduces macrophage-driven inflammation, exerts beneficial effects on adipose remodelling, suppresses adipose tissue inflammation, and has atheroprotective effects on endothelial signalling. However, the current literature also suggests disadvantageous effects of GIP in specific models with proinflammatory signals and worsening of insulin resistance.^13^ This shows that underlying mechanisms are still not completely understood, and further studies such as the present one are needed to close this gap in knowledge regarding the role of GIP within the CV system.

Comparing a GIP pathway-specific score with a polygenic BMI score provides a simple but effective tool to differentiate between pathway-related effects and extent of weight change. GIP-mediated weight loss may provide beneficial CV effects that general BMI change does not fully reflect, as GIP/GIPR signalling was associated with lower risks of MACE and heart failure compared with the polygenic BMI score. Further analyses indicate that traditional CV risk factors explain only some part of this association. This is consistent with CV outcomes in SELECT, where beneficial CV effects of semaglutide were demonstrated to be independent of weight and glucose reduction, potentially mediated by anti-inflammatory effects, as strong reductions in hsCRP were observed.^8^ This interpretation also aligns with positive effects of incretin mimetics in heart failure, as obesity and systemic inflammation are key elements in HFpEF pathophysiology.

While GLP-1RAs are guideline recommended for CV risk reduction in T2DM, protective effects are also present in individuals with overweight or obesity and established ASCVD without diabetes, which is why preventive strategies move beyond glycaemia.^8,14^ Additional CVOTs addressing the twincretin axis (GLP-1 and GIP) in patients with obesity and ASCVD are ongoing (SURMOUNT-MMO, NCT05556512; MARITIME-CV, NCT07037433). Recent evidence showed that dual activation of the GLP-1R and GIPR by tirzepatide leads to substantial weight loss.^9^ However, combining activation of the GLP-1R and inhibition of the GIPR with maridebart cafraglutide also strongly reduced body weight.^15^ The current study by Emanuelsson *et al*. provides important novel findings, but we have to see the outcome data if antagonism or agonism of the GIPR could improve CV prognosis in patients with obesity and established ASCVD.

The observations of the study by Emanuelsson *et al*. should be interpreted in the context of its limitations.^12^ Mendelian randomization only suggests but does not prove causality since some residual genetic confounding may remain. Also, most participants are of European ancestry, which may limit generalizability. Importantly, the present genetic approach does not address the GLP-1R pathway, which is part of the tirzepatide and maridebart cafraglutide molecules, limiting assessment of GLP-1-independent effects of GIP. However, it is important to note that genetic variants in the GLP1R gene that are significantly associated with BMI and/or glycaemia at a level suitable for use in instrumental variable analyses have not been described yet. Nevertheless, these limitations reflect the state of the research area itself, rather than weaknesses of the analysis.

In summary, the study by Emanuelsson *et al*. adds a missing piece to the puzzle to further deciphering the role of GIP in current developments of incretin mimetics. The genetic evidence highlights CV benefits that are not fully explained by weight loss or glycaemic control, supporting GIP as a contributor to CV risk reduction beyond traditional risk factors. Besides established benefits of incretin mimetics in T2DM, in individuals with obesity but without T2DM, and emerging data in HFpEF, these genetic findings provide a rationale for CVOTs addressing the GIP pathway. This is of importance, as an unmet need remains for therapeutic strategies in obesity management. Effective treatments that can be introduced in clinical routine to prevent progression of the CKM syndrome in its early phases are necessary to target obesity as a disease. The aim is not only to achieve weight loss but also to improve outcomes in high CV risk individuals. Incretin-based strategies move in this direction, but broader development and implementation are required. Ongoing CVOTs will shed more light on this, as further agents such as triple agonists and oral formulations advance. For now, the study of Emanuelsson *et al*. is an important step forward into the incretin era.

## References

[1] GBD 2021 Risk Factors Collaborators . Global burden and strength of evidence for 88 risk factors in 204 countries and 811 subnational locations, 1990–2021: a systematic analysis for the Global Burden of Disease Study 2021. Lancet 2024;403:2162–203. 10.1016/S0140-6736(24)00933-4 38762324 PMC11120204

[2] Whitlock G, Lewington S, Sherliker P, Clarke R, Emberson J, Halsey J, et al Body-mass index and cause-specific mortality in 900 000 adults: collaborative analyses of 57 prospective studies. Lancet 2009;373:1083–96. 10.1016/S0140-6736(09)60318-4 19299006 PMC2662372

[3] Ndumele CE, Rangaswami J, Chow SL, Neeland IJ, Tuttle KR, Khan SS, et al Cardiovascular–kidney–metabolic health: a presidential advisory from the American Heart Association. Circulation 2023;148:1606–35. 10.1161/CIR.0000000000001184 37807924

[4] Look AHEAD Research Group; Wing RR, Bolin P, Brancati FL, Bray GA, Clark JM, et al Cardiovascular effects of intensive lifestyle intervention in type 2 diabetes. N Engl J Med 2013;369:145–54. 10.1056/NEJMoa1212914 23796131 PMC3791615

[5] Ussher JR, Drucker DJ. Glucagon-like peptide 1 receptor agonists: cardiovascular benefits and mechanisms of action. Nat Rev Cardiol 2023;20:463–74. 10.1038/s41569-023-00849-3 36977782

[6] Lee MMY, Sattar N, Pop-Busui R, Deanfield J, Emerson SS, Inzucchi SE, et al Cardiovascular and kidney outcomes and mortality with long-acting injectable and oral glucagon-like peptide 1 receptor agonists in individuals with type 2 diabetes: a systematic review and meta-analysis of randomized trials. Diabetes Care 2025;48:846–59. 10.2337/dc24-1533 40156846

[7] Butler J, Shah SJ, Petrie MC, Borlaug BA, Abildstrøm SZ, Davies MJ, et al Semaglutide versus placebo in people with obesity-related heart failure with preserved ejection fraction: a pooled analysis of the STEP-HFpEF and STEP-HFpEF DM randomised trials. Lancet 2024;403:1635–48. 10.1016/S0140-6736(24)00469-0 38599221 PMC11317105

[8] Lincoff AM, Brown-Frandsen K, Colhoun HM, Deanfield J, Emerson SS, Esbjerg S, et al Semaglutide and cardiovascular outcomes in obesity without diabetes. N Engl J Med 2023;389:2221–32. 10.1056/NEJMoa2307563 37952131

[9] Aronne LJ, Horn DB, Roux CWI, Ho W, Falcon BL, Valderas EG, et al Tirzepatide as Compared with Semaglutide for the Treatment of Obesity. N Engl J Med 2025;393:26–36. 10.1056/NEJMoa2416394 40353578

[10] Packer M, Zile MR, Kramer CM, Baum SJ, Litwin SE, Menon V, et al Tirzepatide for heart failure with preserved ejection fraction and obesity. N Engl J Med 2025;392:427–37. 10.1056/NEJMoa2410027 39555826

[11] Krüger N, Schneeweiss S, Fuse K, Matseyko S, Sreedhara SK, Hahn G, et al Semaglutide and tirzepatide in patients with heart failure with preserved ejection fraction. JAMA 2025;33:1255–66. 10.1001/jama.2025.14092 PMC12400167 40886075

[12] Emanuelsson F, Nordestgaard BG, Benn M. Genetic variants of glucose-dependent insulinotropic polypeptide (GIP) signalling as proxy for body weight reduction and cardiovascular risk. Eur Heart J 2026;47:2800–10. 10.1093/eurheartj/ehaf779 PMC13247186 41056187

[13] Müller TD, Adriaenssens A, Ahrén B, Blüher M, Birkenfeld AL, Campbell JE, et al Glucose-dependent insulinotropic polypeptide (GIP). Mol Metab 2025;95:102118. 10.1016/j.molmet.2025.102118 40024571 PMC11931254

[14] Marx N, Federici M, Schutt K, Muller-Wieland D, Ajjan RA, Antunes MJ, et al 2023 ESC guidelines for the management of cardiovascular disease in patients with diabetes. Eur Heart J 2023;44:4043–140. 10.1093/eurheartj/ehad192 37622663

[15] Jastreboff AM, Ryan DH, Bays HE, Ebeling PR, Mackowski MG, Philipose N, et al Once-monthly maridebart cafraglutide for the treatment of obesity—a phase 2 trial. N Engl J Med 2025;393:843–57. 10.1056/NEJMoa2504214 40549887

